# Network analysis of global tobacco control collaboration: data from the World Conference on Tobacco or Health (WCTOH)

**DOI:** 10.1186/s12889-017-4234-7

**Published:** 2017-04-20

**Authors:** Scott J. Leischow, Janet Okamoto, Scott McIntosh, Deborah J. Ossip, Harry A. Lando

**Affiliations:** 1 Mayo Clinic, 13400 E. Shea Blvd, Scottsdale, AZ USA; 2 Department of Public Health Sciences, University of Rochester Medical Center, Rochester, USA; 3 University of Minnesota, Minneapolis, USA

**Keywords:** FCTC, Global tobacco control, Network analysis, Collaboration

## Abstract

**Background:**

The World Conference on Tobacco or Health (WCTOH) is held every three years to foster communication and collaboration on global tobacco control. Very little is known about the nature of interactions between WCTOH attendees and their linkages to tobacco control organizations, so knowing this information could help improve tobacco control efforts.

**Methods:**

At the 2015 WCTOH, we implemented an online survey to assess barriers to global tobacco control activities, which information sources they use for tobacco control information, and with whom they interact regarding tobacco control.

**Results:**

A total of 169 respondents completed the survey, with responses from all six World Health Organization (WHO) regions. Respondents worked in all areas of tobacco control; the most common were research (29.2%) and patient care/treatment (23.3%). The top barriers faced regarding tobacco control activities were: funding is weak (56.8%), government commitment (45.0%), tobacco industry interference (43.8%), and lack of coordination (34.3%). The network analysis identified Framework Convention Alliance (FCA) and Society for Research on Nicotine and Tobacco (SRNT) as the two most prominent groups that people belonged to and where they went to exchange information and best practices. Important regional and country specific groups also appear to be growing, such as the African Tobacco Control Alliance (ATCA) and the Argentinian Association of Tabacology (ASAT).

**Discussion:**

Mapping and better understanding the global tobacco control network is important for informing knowledge exchange and best practices, particularly as increasing attention is being focused on global tobacco control efforts in low- and middle-income countries in particular.

**Conclusions:**

The present study demonstrates that even a subsample of the WCTOH shows considerable collaboration. The full WCTOH network should be mapped in order to foster greater collaboration that has the the potential to improve global tobacco control efforts.

## Background

Since 1967, the global tobacco control community has assembled every three years for the World Conference on Tobacco or Health (WCTOH) to disseminate new knowledge about the risks of tobacco use and exposure, and to explore new tobacco control approaches that are effective [1]. The WCTOH has been a consistent venue for the tobacco control community to function as a network, and to expand itself as a network with the goal of eliminating tobacco caused morbidity and mortality. Initially, tobacco control efforts were largely the interest of high-income countries, primarily in Europe, North America, and Australia, but expanded surveillance and research showed the dramatic impact of tobacco in low- and middle-income countries, and tobacco control efforts have since expanded rapidly [1]. Many organizations and governments increased their support for global tobacco control efforts, including support for the WCTOH to share new ‘learnings’. This increased support has allowed the WCTOH to become more global by expanding inclusion of tobacco control efforts in low and middle-income countries.

The tobacco control network has expanded to include a broad array of committed partners, from children in low-income countries who recognize the need to prevent their peers from becoming addicted, to scientists who have clearly demonstrated to the world the associations between tobacco exposure and cancer, to world leaders who shape global policy at the World Health Organization. At the WCTOH, this array of tobacco control advocates come together to share what has been learned in an effort to enhance tobacco control progress in homes, communities, states, countries and in global policy.

The success of the tobacco control movement is perhaps best represented by a major global public health landmark: the Framework Convention on Tobacco Control (FCTC) [2]. In 1999, many organizations collaborated to create the Framework Convention Alliance (FCA) with the common goal of developing and implementing a global treaty to “adopt a comprehensive range of measures designed to reduce the devastating health and economic impacts of tobacco” [2]. The FCA organizations rapidly developed a set of evidence-based guidelines, the FCTC, to maximize global tobacco control efforts. In 2005, the FCTC became the first-ever ratified public health treaty, and this treaty is the culmination of research and tobacco control practice that serves as a blueprint for guiding global tobacco control efforts. With 180 countries party to the FCTC as of March 2015 [3], the ‘Articles’ of this treaty call for countries that have ratified the treaty to implement price and tax changes to reduce demand for tobacco, to assure reductions in exposure to secondhand smoke, to reduce advertising and to control packaging and labeling to reduce use, and to help smokers to quit, along with several additional evidence-based tobacco control priorities that together, when fully implemented, could lead to dramatic reductions in tobacco use.

Indeed, one section of the FCTC includes language stating that the tobacco control community should work together to achieve “scientific and technical cooperation and communication of information”, and it includes three distinct Articles that address coordination of research and surveillance, regular reporting of progress on implementation of the FCTC, and cooperation on efforts to optimize capacity to best assure implementation of the FCTC (see Table 1). Thus, the FCTC reinforces and strengthens efforts that began with the first WCTOH in 1967. It seeks to assure that the tobacco control community comes together to maximize accomplishing the FCTC goals in the most effective way.

**Table 1:** FCTC articles addressing scientific and technical cooperation and communication of information

FCTC Article	Description
20	Under Article 20, Parties undertake to develop and promote national research and to coordinate research programmes internationally, as well as to establish and strengthen surveillance for tobacco control and to promote exchange of information in relevant fields.
21	Parties are required, under Article 21, to submit to the COP, through the Convention Secretariat, periodic reports on implementation of the Convention. The COP determines the frequency and format of such reports. In 2010, COP4 adopted a decision to introduce a biennial reporting cycle that began in 2012.
22	Article 22 requires Parties to cooperate directly or through competent international bodies to strengthen their capacity for implementing obligations arising from the Convention.

Since 1967, the WCTOH has helped to document the great strides that have been achieved in global tobacco control, and has exemplified the principles that led to the inclusion of Articles 20–22 that call for capacity building and exchange of information on ‘what works’ in tobacco control. A challenge of the WCTOH, however, is that it is held only once every three years, and there is no formal infrastructure in place to foster communication between those conferences. Moreover, even though the goal of the WCTOH is facilitating networking between attendees for knowledge sharing, there has been limited effort to understand the nature of that networking, or to assess in what way the WCTOH fosters communication and collaboration between conferences. Research designed to understand and strengthen tobacco control communication and collaboration by analyzing tobacco control networks has been identified as an important focus [4, 5]. The current research team has been active in the identification and analysis of other collaborative tobacco control networks, using methods applicable for filling this gap.

As part of the planning meetings in advance of the 16th WCTOH, which took place in March 2015 in Abu Dhabi, the Executive Committee of the WCTOH Scientific Program Committee expressed interest in better understanding the communication and collaboration between conference attendees. This led to the development and implementation of a ‘proof of concept’ pilot study survey designed to help document international tobacco control communication and collaboration among individuals and organizations in attendance at the WCTOH, with the eventual goal of fostering a functional communication and collaboration network to improve global tobacco control efforts. Thus, the aim of the present study was to assess and map the communication and collaboration between a subset of individuals and organizations that attended the WCTOH in order to better understand the tobacco network, with the goal of using that information to expand analysis of the network and foster sharing of information to improve global tobacco control initiatives.

## Methods

In consultation with the WCTOH Scientific Program Executive Committee, and building on our earlier work [6–13], survey questions were developed, pretested and pilot tested in an iterative manner. The resulting 22 survey items assessed respondents’ background in tobacco control (e.g., years in the field, type of organization represented, amount of time dedicated to tobacco control, role in tobacco control efforts) as well as tobacco control activities (e.g., FCTC articles worked on, participation in tobacco control groups/organizations, and involvement in various activities such as research and training). In addition, to better understand each respondent’s collaboration network, respondents were asked to list the top five organizations/individuals who are either within or outside of their country with whom they work on tobacco control initiatives, as well as how frequently they work with those organizations/individuals. These network questions were intended to identify smaller regional- and country-level networks and infrastructures. In order to assess knowledge sharing, respondents were asked how often they used various sources of information about tobacco control issues, which included: tobacco control journals, other academic journals, national or regional conferences, international conferences, websites/internet searches, newsletters, listservs or blogs, and communications with colleagues/experts. Respondents were also asked to report barriers faced in their country or region regarding tobacco control activities, such as tobacco industry interference, lack of government commitment, weak funding, low tobacco taxes, and lack of training opportunities in tobacco control. Finally, basic demographic information was collected and included organization, city, country, language, gender, age, highest academic degree, and primary academic training.

Pilot testing, designed to improve clarity and specificity of the survey questions, was accomplished at the University of Rochester, and was led by one of the study co-authors (SM). This process involved pretesting multiple iterative drafts of the instrument, initially in document form, then in its online form, to elicit ongoing refinement of existing items and inclusion of additional items. Specific feedback was elicited from project team members and colleagues with knowledge of content in at least some of the domains. Items, as well as online formatting options, were improved until a final version of the instrument was agreed upon.

An additional goal of the pilot/pretesting process was to assure that time to survey completion would not serve as a barrier, with the final version timed on average at 10–15 min for completion. The survey was only available in English for multiple reasons: (1) this was a pilot study, (2) resources were limited, and (3) the official language of the WCTOH is English, so it seemed likely that most attendees would have at least some English-language proficiency. Although the Executive Committee and survey team recognized that an English-only survey would serve as a limitation and result in less than optimal completion, the goal of the pilot study was to begin to assess whether communication networks would emerge that are relevant to improving communication and collaboration. The pilot study also would serve as a foundation for both future expanded survey implementation and as a means of encouraging communication and collaboration between WCTOH conferences.

Once the survey was pilot tested and approved by the Executive Committee, it was converted to a final online format, and a web link for the survey was created. Prior to the WCTOH, the online survey link was featured on the WCTOH website, included in WCTOH promotional emails, and was maintained throughout the conference in March 2015. Additional dissemination of the survey link occurred via various international tobacco control listservs. No additional efforts to increase rates of completion were implemented, such as requiring completion as part of the registration process, because of the pilot nature of the project.

## Data analysis

Data were downloaded from the online survey instrument. Data cleaning, organization, and descriptive statistics were calculated using Stata 11 [14]. Basic network metrics were calculated using UCINet 6 [15]. Visualizations were done using the Gephi software for network analysis [16] and UCINet 6. Organization and country names were maintained and presented in the network diagrams, while no individual names of tobacco control colleagues identified in surveys were displayed.

## Results

The number of respondents who submitted the questionnaire was 169. While more than 3000 attended the conference, it is not possible to accurately identify the denominator so that a response ratio could be determined because it is not certain how many attendees actually received the request to complete the instrument. As indicated, announcements about the survey were posted on the WCTOH website, but we do not know how many people saw those announcements.

Respondents were slightly more likely to be males than females (56% vs. 44%), and the primary language spoken was English. While the survey was provided in English, 14.9% of respondents spoke Spanish; 3.7% Chinese; 17.4% French; 5.6% Arabic; 30.4% another language. All WHO Regions were represented, though with considerable variability. The largest representation was from the Americas (34.2%), followed by Europe (19.3%), Southeast Asia (16.2%), Western Pacific (13.7%), Africa (10.6%) and Eastern Mediterranean (6.2%). Academic training varied, with 27% reporting prior training in medicine, 21% in social sciences, 18% in public health, 4% each in biological sciences and law, and 26% in ‘other’. The average number of WCTOH conferences that respondents attended was 2.12 (SD = 2.92, range = 0–15), and the average duration working in tobacco control was 13.97 years (SD = 9.79, range = 0–40).

When asked about which tobacco control groups/organizations they belonged to, respondents most frequently listed (out of 179 unique groups reported): Framework Convention Alliance (FCA) (26), Society for Research on Nicotine and Tobacco (SRNT) (22), Global Bridges (GB) (10), and Association for the Treatment of Tobacco Use and Dependence (ATTUD) (10). The majority of groups listed by respondents were country specific or regional groups, such as the Cameroon Coalition to Counter Tobacco, Bangladesh Anti-Tobacco Alliance, Rajasthan Coalition for Tobacco Control, and Smokefree Tasmania. Other groups mentioned, shown in Table 2, included: The Union, Asia-Pacific Conference on Tobacco or Health (APACT), European Network for Smoking and Tobacco Prevention (ENSP), Africa Tobacco Control Alliance (ATCA), and the International Network of Women Against Tobacco (INWAT).

**Table 2:** Top organizations identified as collaborators

Top ten tobacco control groups/organizations respondents belong to/participate in:	Top ten organizations respondents work with on tobacco control:
FCA	The Union
SRNT	WHO
Global Bridges	FCA
INWAT	CTFK
ENSP	SRNT
ATTUD	American Cancer Society
Africa Tobacco Control Alliance (ATCA)	U.S. CDC
South East Asia Tobacco Control Alliance (SEATCA)	Ministry of Health (common name but from various countries)
Asia Pacific Conference on Tobacco or Health (APACT)	Global Bridges
Action on Smoking or Health (ASH)	FDA Center for Tobacco Products

**Fig. 1:**
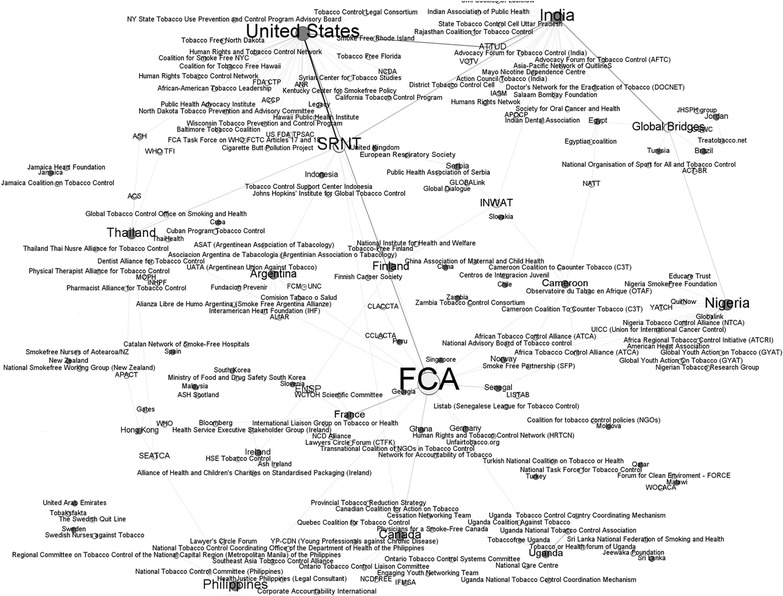
Network of tobacco control groups/organizations by country. Legend: *light grey nodes* = organization; *dark grey nodes* = country. Nodes are sized on in-degree centrality, or the number of incoming ties to the node. Ties are colored by weight, with *darker lines* indicating more respondents from a country naming a group/organization

The network map (Fig. 1) showing group membership relative to country of origin provides valuable insight into communication across countries and organizations. Countries are shown in dark gray, and organizations in white, and the size of the circle indicates the number of ties to that country or organization. Thus, the organization with which the largest number of respondents interact is the Framework Convention Alliance (FCA). The Society for Research on Nicotine and Tobacco (SRNT) and Global Bridges were also central nodes in this network. Respondents from the United States represented the largest node, but India, Canada, Nigeria, the Philippines, and Thailand also represented sizable nodes in the network. However, despite the clear linkages to large international organizations shown in Fig. 1, organizations linked to specific countries demonstrate that considerable tobacco control activity is locally and regionally oriented.

**Fig. 2:**
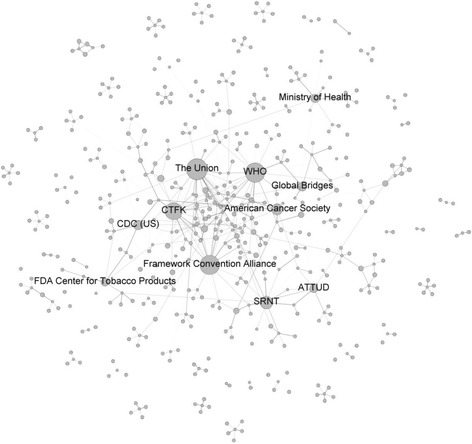
Most prominent nodes who respondents reported they most commonly worked with on tobacco control. Nodes are sized on in-degree centrality, or the number of incoming ties to the node. Ties are colored by how frequently respondents worked with an organization/individual with *darker lines* indicating more frequent contact

Figure 2 shows a diagram of the most prominent nodes in the network of respondents who reported they most commonly worked with on tobacco control. In this map, the FCA is again very central, but The Union is now clearly a very central node, along with the WHO, and the Campaign for Tobacco-Free Kids (CTFK). There are many organizations and individuals that play a central role in this network, as indicated by their position in the map, even if they do not have as many clear ties to them (e.g., World Lung Foundation). And there appear to be organizational units that are isolates, i.e., not linked to other groups, and those organizations and individuals represent opportunities for building linkages if in fact no current linkages exist.

The survey also asked respondents to identify which FCTC articles they work on the most (respondents were allowed to select all that apply). Warning about the dangers of tobacco (Articles 11 & 12) was reported by 55.5% of the respondents to be the FCTC priority on which they dedicated most of their time, followed by 52.4% on tobacco treatment (Article 14), 45.1% on protection from secondhand smoke (Article 8), 40.2% on monitoring tobacco use and prevention policies (Article 20), 32.9% on enforcing bans on advertising and promotion of tobacco (Article 13), 22% on raising taxes (Articles 6 & 15), and 17.7% on other Articles.

**Fig. 3:**
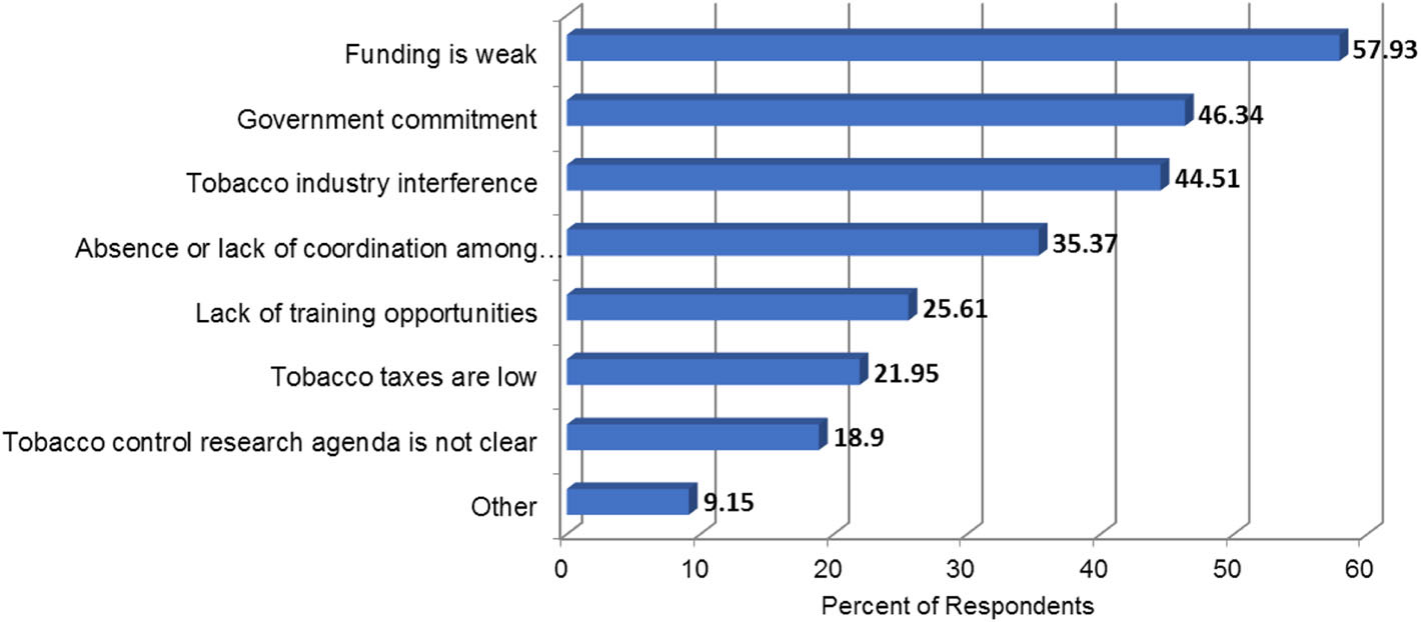
Barriers for implementation of tobacco control structure and function

Respondents were also asked to identify the top three barriers in their country or region for implementation of tobacco control structure and function. As indicated in Fig. 3, funding is considered the greatest barrier by respondents (57.9%), followed by lack of government commitment (46.3%), tobacco industry interference (44.5%), absence of coordination among different groups (35.4%), lack of training opportunities (25.6%), low tobacco taxes (22%) and an unclear tobacco control research agenda (18.9%). Other barriers reported by respondents included: high levels of staff turnover, lack of time due to under-staffing, low awareness among medical staff, and weak control or legislation.

## Discussion

This first-ever analysis of WCTOH attendees through a pilot network survey provides an initial look at global tobacco control communication and collaboration, along with barriers and priorities that can help to identify future opportunities for achieving the goals articulated in the FCTC. Overall, the organizational network analysis indicated that the FCA was clearly the most central organization identified by respondents. This is not surprising given its breadth and scope of activities and members, but this clearly indicates that the FCA is a central node that is highly relevant for knowledge flow for those who responded to the survey. Other organizations that emerged as key nodes were the WHO, SRNT, INWAT, ENSP and Global Bridges. These organizations are networks in their own right, so in effect this analysis is assessing networks of networks, and suggests that organizations that focus on global health goals do, in fact, function as key points of information exchange and/or collaboration.

The country by organization analysis depicted in Fig. 1 also demonstrated that some respondents did not indicate linkages with any organizations outside of their own countries (e.g., Netherlands and Jamaica). It is possible that those country/organization relationships are isolated and unconnected, which would suggest opportunities for global outreach. However, it is also possible that inclusion of a broader range of respondents from those countries could have changed the map. Nonetheless, this country by organization network map indicates that, overall, responders tended to have a large number of within and between country linkages, thus demonstrating the depth and breadth of the tobacco control community even as represented by this limited sample.

This process of assessing networks of networks is critical when considering the complex tobacco control ecosystem because these subnetworks were typically created to achieve specific goals, e.g., to focus on a specific FCTC Article. By assessing ways that these focused networks interact with each other networks, it will be possible to obtain a clearer perspective on the overall ‘health’ of the tobacco control environment, and the extent to which collaborative efforts can address the FCTC Articles in their totality. That is, by understanding the various organizations and their communication and collaboration within and between each other, it will be possible to better identify areas of strength and success in tobacco control, but also to identify potential ‘holes’ in the global tobacco control environment that need to be addressed to better achieve the FCTC goals.

Indeed, the networks identified in this analysis help to characterize, in part, the nature of the global tobacco control system’s strengths and weaknesses. For example, one of the characteristics of the tobacco control network is that it is a ‘federated system’, or ‘system of systems’, i.e., there is no one organization that controls the network structure and function [17–19]. A federated system has no top down decision structure and function, and depends a great deal on shared governance, the development of communication and collaboration structures that assure both good flow of new knowledge as well as adaptability to change, and a clear vision of the overall goals that are to be achieved so that the many partners can work their way toward those goals. Federated systems are in some respects very inefficient because they depend on the creation of infrastructures, such as the WCTOH, to help the many different organizations to revisit their priorities, share new knowledge on what works and doesn’t, and in general to strive to achieve the shared goals of their respective organizations (recognizing that there are considerable differences in the extent to which those organizations will prioritize addressing one or more of the FCTC articles).

The data collected in this small pilot survey make clear that considerable communication and collaboration is occurring across the global tobacco control community, but just as clearly indicates that considerable challenges remain. For example, participants (a) identified funding as a major challenge, (b) noted that the tobacco industry interferes with their efforts and (c) have concerns about lack of government commitment. These three challenges are inter-related, and reflect the reality that has always existed regarding tobacco control efforts: the tobacco industry and the companies that rely on the tobacco industry (e.g., convenience stores) generate very large profits and provide a large amount of revenue to government coffers, so reducing or eliminating that revenue by increasing tobacco control efforts (e.g., funding) may not be viewed as a priority by governments with little revenue from other sources. Moreover, there are many studies showing that the tobacco industry has interfered with tobacco control efforts, which is why one of the FCTC priorities is reducing tobacco industry influence (reference for FCTC).

However, addressing these challenges represents intermediate to long-term goals that will take considerable coordination and focus to achieve. Importantly, the observed barrier of a lack of coordination among different groups is something that the tobacco control community can address in a way that does not require massive quantities of funding or structural change at the government level. Moreover, these data suggest that communication and collaboration can be expanded and improved between the WCTOH conferences that occur every three years, and perhaps foster improvements in knowledge flow that can speed tobacco control efforts. The network analysis in this small project is a first step in better understanding this system of systems in tobacco control, with the goal of subsequently expanding the analysis to include the full range of tobacco control individuals and organizations globally, and using that information to better achieve the FCTC article goals that mandate improving knowledge flow so that advances in tobacco control can be achieved at a faster pace.

While this study has identified valuable new information on the global tobacco control ‘system of systems’, including some strengths and weaknesses of that system, the results also suggest that expanded network analyses are warranted in order to obtain a more complete picture of the global tobacco control network. Only by better understanding the network, and how it functions, can that network be optimized to achieve its tobacco control goals. This recognition of the need for expanded analyses reflects one of the clear weaknesses of the current study, its small sample size. Because this survey was a convenience sample, it represented fewer than 10% of the total number of WCTOH attendees. Generalizability is further limited by the fact that the global tobacco control community is much larger than those who to attend the WCTOH. Future research should target a larger sample of those who attend the WCTOH, which could be accomplished by making completion of the survey a requirement for registration, and by implementing the survey through other regional and national tobacco control groups.

Additional limitations are that the survey was only provided in English, which likely limited the numbers of respondents even though presentations at the WCTOH were only in English. In addition, while the WCTOH conference is a well-attended international conference, certain groups, countries and regions, particularly LMICs, may be underrepresented in the sample. Lastly, because participants were informed that their name or the name of their organization could be publicly indicated, it is possible that some people chose to refrain from participating for that reason. Nonetheless, this study has provided a first snapshot of communication and collaboration among a sample of WCTOH attendees, and provides a methodology for replication with a broader sample of WCTOH registrants in 2018 (and perhaps earlier). Results can guide establishment of an infrastructure to foster expanded communication and collaboration between WCTOH conferences in order to optimize the global tobacco control ‘system of systems’.

## Conclusions

The primary aim of this study was to assess communication and collaboration among and between WCTOH attendees and organizations, and to generate a network map of those local and global interactions, given the goal of the World Conference on Tobacco or Health (WCTOH) is to serve as a forum for communication and collaboration. The present study demonstrates that even a subsample of the WCTOH shows considerable collaboration. Moreover, several international organizations clearly serve as knowledge brokers for tobacco control efforts across countries, which has relevance for understanding which organizations have the potential to reach many different individuals and organizations as a result of their central position in the network. These promising results suggest that the full WCTOH network should be assessed because of the potential to both understand collaboration but also to use that network information to enhance dissemination of evidence-based practices that have the potential to improve tobacco control efforts.
